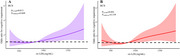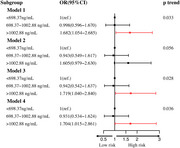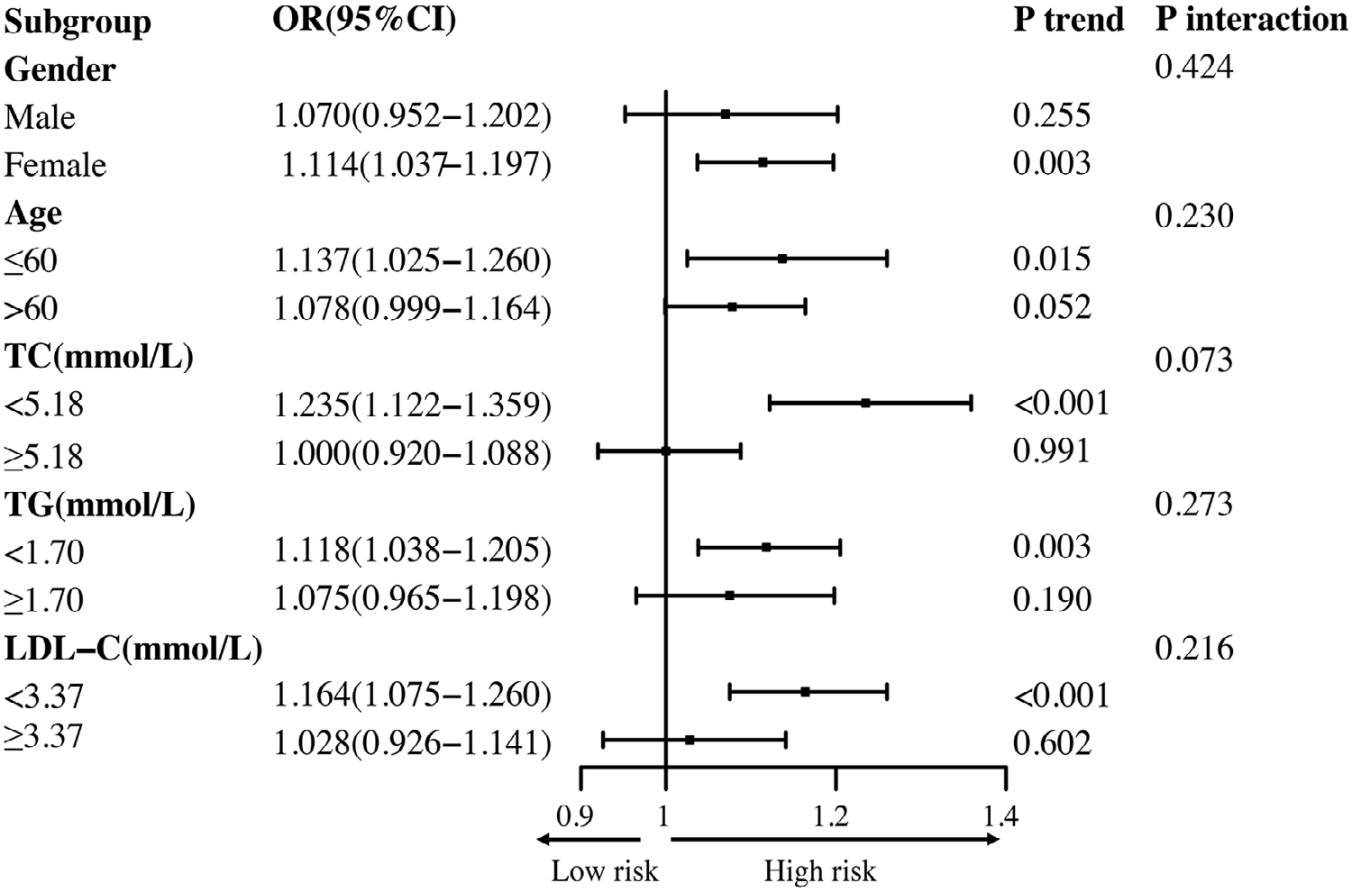# The relationships between plasma oxidized LDL and cognitive impairment: a population‐based cross‐sectional study in China

**DOI:** 10.1002/alz70860_101798

**Published:** 2025-12-23

**Authors:** Yi Zhao, Liangjun Dang, Suhang Shang, Shan Wei, Chen Chen, Jingyi Wang, Qiumin Qu, Jin Wang

**Affiliations:** ^1^ The First Affiliated Hospital of Xi’an Jiaotong University, Xi’an, shaanxi, China; ^2^ The First Affiliated Hospital of Xi'an Jiaotong University, Xi'an, Shaanxi, China; ^3^ Huxian Hospital of Traditional Chinese Medicine, Xi'an, Shaanxi, China

## Abstract

**Background:**

It has been reported that plasma oxidized low‐density lipoprotein (ox‐LDL) contributes to the atherosclerosis and triggers cardiovascular and cerebrovascular events. However, the relationship between plasma ox‐LDL level and cognitive impairment had not been determined. This study investigated the association between plasma ox‐LDL level and cognitive impairment among middle‐aged and older population in China.

**Method:**

This was a community population‐based cross‐sectional study. A total of 1,406 participants (≥ 40 years) were recruited from a rural village in Xi’an, China using cluster‐sampling method. The cognitive function was evaluated using the Mini‐Mental State Examination (MMSE) and a battery of neuropsychological assessment, and cognitive impairment was diagnosed based on the criteria for mild cognitive impairment and dementia. Plasma ox‐LDL levels were measured using ELISA. Restricted cubic spline analysis, multiple logistic regression, trend analysis and subgroup analysis wereperformed to investigate the relationships between plasma ox‐LDL and cognitive impairment.

**Result:**

A total of 1,406 participants were included, of whom 112 (8.0%) met the criteria for cognitive impairment. Restricted cubic spline analysis did not identify a non‐linear relationship between ox‐LDL level and cognitive impairment (*P*_non‐linear_=0.3167). Multivariable logistic regression showed that the incidence of cognitive impairment increased by 9.3% per 100 unit increase in the ox‐LDL level (OR=1.093, 95% CI: 1.031‐1.160, *p* = 0.003). Cognitive impairment risk was 1.704‐fold higher in the highest ox‐LDL tertile compared to the lowest ox‐LDL tertile (OR=1.704, 95% CI: 1.015–2.861, *p* = 0.044). Interaction analysis revealed sex, age, total cholesterol, triglycerides, and low‐density lipoprotein did not significantly modify the association between ox‐LDL levels and cognitive impairment.

**Conclusion:**

Elevated ox‐LDL levels are associated with a higher likelihood of cognitive impairment among Chinese adults aged ≥40 years. However, the causal relationship between plasma ox‐LDL levels and cognitive impairment needs to be clarified further.